# Complete Genome Sequence of an Alkane Degrader, *Alcanivorax* sp. Strain NBRC 101098

**DOI:** 10.1128/genomeA.00766-14

**Published:** 2014-08-14

**Authors:** Takamasa Miura, Keiko Tsuchikane, Mitsuru Numata, Maiko Hashimoto, Akira Hosoyama, Shoko Ohji, Atsushi Yamazoe, Nobuyuki Fujita

**Affiliations:** Biological Resource Center, National Institute of Technology and Evaluation, Shibuya-ku, Tokyo, Japan

## Abstract

*Alcanivorax* sp. strain NBRC 101098 was isolated from seawater in Japan. Strain NBRC 101098 is able to degrade various types of *n*-alkanes. Here, we report the complete genome of strain NBRC 101098.

## GENOME ANNOUNCEMENT

The genus *Alcanivorax* is a group of alkane-degrading bacteria in the sea, and this degradative activity is expected to be applied to the bioremediation of oil-polluted seawater (1). *Alcanivorax* sp. NBRC 101098 was isolated from Sea of Japan in Ishikawa Prefecture, Japan. Strain NBRC 101098 has the highest degradation activity on crude oil, C_16_-*n*-alkane, and pristane compared with that of other *Alcanivorax* sp. strains in the NBRC culture collection (NBRC 101092, NBRC 101094, NBRC 101095, NBRC 101096, NBRC 101097, NBRC 102021, NBRC 102022, NBRC 102023, NBRC 102024, NBRC 102027, and NBRC 102028). The 16S rRNA gene sequence analysis showed 99.9% similarity to that of *Alcanivorax borkumensis* SK2^T^ (Y12579) (2).

The genome of strain NBRC 101098 was sequenced by single-end sequencing with 454 GS FLX Titanium (Roche, Basel, Switzerland) and paired-end sequencing with the Illumina HiSeq 1000 sequencing system (Illumina, San Diego, CA, USA). The 454 GS FLX Titanium single-end data (89,178 reads, 62,663,291 bp) and the Illumina paired-end data (2,000,000 reads, 200,000,000 bp) were assembled with Newbler version 2.6 (Roche). The genome closure was accomplished by manual adjustment of the assembly and additional Sanger sequencing of targeted PCR products. The complete sequence of the chromosome was analyzed for predicting protein-coding, tRNA, and rRNA genes using Microbial Genome Annotation Pipeline (MiGAP) (http://www.migap.org/), which internally utilizes MetaGeneAnnotator (3), RNAmmer (4), tRNAScan-SE (5), and BLAST (6). The functional annotations of predicted protein-coding genes were assigned based on UniProt (http://www.uniprot.org/) and InterPro (https://www.ebi.ac.uk/interpro/). The genome of strain NBRC 101098 consists of a 3,095,417-bp circular chromosome with 54.7% G+C content, and it contains 2,824 coding sequences (CDSs), 9 copies of rRNA genes, and 42 tRNA genes.

A calculation of the average nucleotide identity (ANI) was also performed between the genome sequences of NBRC 101098 and those of 4 type strains, i.e., *A. borkumensis* SK2^T^ (accession no. AM286690), *Alcanivorax hongdengensis* A-11-3^T^ (accession no. AMRJ01000000), *Alcanivorax dieselolei* B5^T^ (accession no. CP003466), and *Alcanivorax pacificus* W11-5^T^ (accession no. AJGP01000000), using the JSpecies program, with default settings for the ANI against BLAST (ANIb) (http://www.imedea.uib.es/jspecies). As a result, the ANI values of strain NBRC 101098 against the other type strains were 99.5%, 74.3%, 71.3%, and 69.1%, respectively, so it is considered that strain NBRC 101098 is classified into the species *A. borkumensis* (7).

Seven genes were predicted as being responsible for the initial hydroxylation step in alkane degradation, including two alkane monooxygenase genes (AS19_01260 and AS19_27640), three cytochrome P450 genes (AS19_02080, AS19_23510, and AS19_24460), and two flavin-binding monooxygenase genes (AS19_01960 and AS19_02920). Those genes are found to be common between the previously sequenced *Alcanivorax* strains, which may reflect their ubiquitous distribution and specialized adaptation to hydrocarbon degradation in the marine environment.

### Nucleotide sequence accession number.

The nucleotide sequence of *Alcanivorax* sp. NBRC 101098 chromosome was deposited in the DDBJ/EMBL/GenBank databases under the accession no. AP014613.
